# Ultra-long coherence times amongst room-temperature solid-state spins

**DOI:** 10.1038/s41467-019-11776-8

**Published:** 2019-08-28

**Authors:** E. D. Herbschleb, H. Kato, Y. Maruyama, T. Danjo, T. Makino, S. Yamasaki, I. Ohki, K. Hayashi, H. Morishita, M. Fujiwara, N. Mizuochi

**Affiliations:** 1 Institute for Chemical Research, Kyoto University, Gokasho, Uji, Kyoto 611-0011 Japan; 2 National Institute of Advanced Industrial Science and Technology (AIST), Tsukuba, Ibaraki 305-8568 Japan

**Keywords:** Sensors and biosensors, Quantum information, Sensors, Qubits

## Abstract

Solid-state single spins are promising resources for quantum sensing, quantum-information processing and quantum networks, because they are compatible with scalable quantum-device engineering. However, the extension of their coherence times proves challenging. Although enrichment of the spin-zero ^12^C and ^28^Si isotopes drastically reduces spin-bath decoherence in diamond and silicon, the solid-state environment provides deleterious interactions between the electron spin and the remaining spins of its surrounding. Here we demonstrate, contrary to widespread belief, that an impurity-doped (phosphorus) n-type single-crystal diamond realises remarkably long spin-coherence times. Single electron spins show the longest inhomogeneous spin-dephasing time ($T_2^ \ast \approx 1.5$ ms) and Hahn-echo spin-coherence time (*T*_2_ ≈ 2.4 ms) ever observed in room-temperature solid-state systems, leading to the best sensitivities. The extension of coherence times in diamond semiconductor may allow for new applications in quantum technology.

## Abstract (web summary)

The coherence times of nitrogen-vacancy centres are key factors influencing their performance in quantum applications. Here the authors show that synthesising phosphorus-doped diamond yields nitrogen-vacancy centres with significantly improved $T_2^ \ast$ and *T*_2_.

## Introduction

Solid-state spins are a leading contender in quantum technology^1^. Systems such as colour centres in diamond^2,3^, silicon carbides^4,5^, rare-earth ions in solids^1,6^, donors in silicon^7–9^, and quantum dots^7–10^ have been investigated thoroughly. Enhancing the inhomogeneous spin-dephasing time ($T_2^ \ast$) and the Hahn-echo spin-coherence time (*T*_2_) is a central issue. The electron spin plays a significant role for quantum sensing and for coherent connectivity with other qubits, such as photons, nuclear spins, and superconducting qubits^11,12^. Therefore, these times define the physical behaviour of the quantum device, and their improvement allows new perspectives for quantum applications. For example, their increase directly improves direct current (DC) and alternating current (AC) sensitivities of nitrogen vacancy (NV) sensors^3,13^, quantum-gate fidelity^7,9^, and quantum-memory times^3,7,8^.

From the viewpoint of material science, enhancements of $T_2^ \ast$ and *T*_2_ have been realised by development of growth techniques to suppress paramagnetic defects^14^, isotope engineering to reduce nuclear spins^3,7,15^, and annealing techniques to remove defects^16^. Regarding diamond, there has been remarkable progress in the quality of single crystal diamond grown by chemical vapour deposition (CVD)^14^. By suppressing impurities and defects, the *T*_2_ of NV centres has been enhanced. For example, *T*_2_ = 0.7 ms is reported, which is the longest *T*_2_ at room temperature among diamond that contains a natural abundance (1.1%) of ^13^C^15,17^. Furthermore, by depleting the ^13^C isotope, the electron spin showed significantly long room-temperature spin-dephasing times ($T_2^ \ast = 100$ μs^18^, 470 μs^2^) and spin-coherence times (*T*_2_ = 1.8 ms in single-crystal^3^ and 2.0 ms in poly-crystal diamond^19^).

In principle, *T*_2_ can be extended closer to *T*_1_ (~6 ms^20^, ~7.5 ms^2^, more details later on); however, this has not been reached yet. The reason considered is the effect of residual paramagnetic defects and/or impurities in the bulk. During the CVD growth^21^ and ion-implantation^22^, it is known that many vacancies are generated. Once they combine, complexes or defect clusters are formed. It is very hard to remove them by annealing techniques, because they are very stable. Therefore, the longest $T_2^ \ast$ and *T*_2_ of single NV centres have been realised by native NV centres created during CVD growth^2,3,19^, while those of NV centres created by ion-implantation and a high annealing temperature have not exceeded the longest ones^16^.

Recently, charging of vacancies suppressed the formation of vacancy complexes by confining implantation defects into a space-charge layer of free carriers created by a thin sacrificial boron-doped p-type diamond layer. After removing this layer by an etching process, the *T*_2_ of the NV centres was improved  at the shallow surface region only ^22^. In their research, intrinsic diamond was used, but n-type conductivity is crucially important because of the stabilisation of the negatively charged state of the NV centres (NV^−^)^23^ and because of the electrical controls used in diamond quantum devices^24–27^.

In the following, by applying a phosphorus-doped n-type CVD diamond growth technique, we demonstrate the longest $T_2^ \ast$ and *T*_2_ ever observed in solid-state systems at room temperature. Utilising the NV centre with the longest *T*_2_, we find, as is expected, that it is the most sensitive single NV centre AC magnetic field sensor. Moreover, by performing noise spectroscopy and *T*_1_ measurements, we show that the result is amenable to further improvement.

## Results

### Sample preparation

The n-type diamond samples A–H were epitaxially grown onto Ib-type (111)-oriented diamond substrates by microwave (MW) plasma-assisted CVD with enriched ^12^C (99.998%) and with phosphorus concentrations ranging from 3 × 10^15^ to 1 × 10^17^ atoms cm^−3^^28^. For all samples, the growth conditions are the same except for the PH_3_/CH_4_ gas ratio to change the phosphorus concentration (see “Methods” section). These growth conditions were chosen so as to realise the highest quality of n-type diamond, with electron mobilities similar to the highest ones reported previously (800 cm^2^ V^−1^ s^−1^ at 300 K^28^, see “Methods” section). The thickness of the resulting layer is in the order of tens of micrometres. We address individual electron spins residing in NV centres with a standard home-built confocal microscope. MW pulses are applied via a thin copper wire, while magnetic fields are induced with a coil near the sample. All experiments were conducted at room temperature.

### Doping concentration comparison

**Fig. 1:**
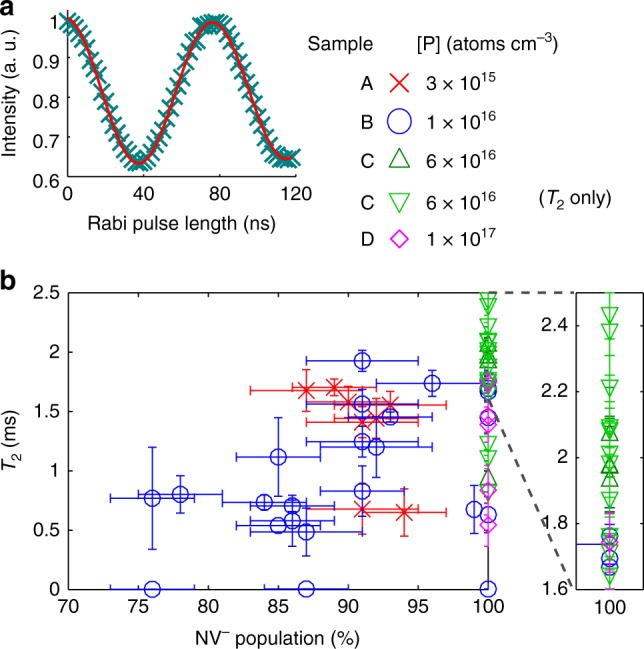
Measuring nitrogen vacancy (NV) centres in phosphorus-doped diamond. **a** Result of Rabi measurement (data with blue crosses, sinusoidal fit with red line, 37% contrast). **b** *T*_2_ vs NV^−^ population for four samples A–D with different doping levels: 3 × 10^15^ (red, crosses), 1 × 10^16^ (blue, circles), 6 × 10^16^ (green, up-pointing triangles have verified NV^−^ population, down-pointing triangles were added to show more statistics for *T*_2_ only, but do likely have the same population), and 1 × 10^17^ atoms cm^−3^ (magenta, diamonds). The error bars indicate standard errors. The top right of the graph is enlarged

In order to compare the doping concentrations, along with Rabi measurements (Fig. 1a), two parameters were measured for a number of NV centres in every sample: the population of the NV^−^ state, and *T*_2_. The NV^−^ population is determined by single-shot charge-state measurements^29^, and *T*_2_ with a Hahn-echo measurement^20^. The results of samples A–D are plotted in Fig. 1b. In the highly doped diamond samples C and D, a charged state of NV^−^ near 100% is realised^23^, while, regardless of the paramagnetic dopant (electron spin 1/2), *T*_2_ can surpass 2 ms. The different local surroundings of each NV centre are considered to be the reason for the scatter of *T*_2_ for each NV^−^ population. NV centres with a higher NV^−^ population show a longer maximum *T*_2_. Moreover, sample C with [P] = 6 × 10^16^ atoms cm^−3^ seems to produce NV centres with longer *T*_2_s than diamond with both smaller and larger phosphorus concentrations, with an average over the first 21 measured NV centres of 〈*T*_2_〉 = 1.8 ms and almost 40% has *T*_2_ > 2.0 ms. This might indicate a competition between a positive effect of phosphorus doping, discussed later, and the negative effect of magnetic noise stemming from phosphorus, and therefore an optimum concentration could exist.

Additionally, we measured *T*_2_ in samples E, F, and G, which have almost the same phosphorus concentration as sample C, and in sample H, which has a larger phosphorus concentration. The population of the NV^−^ state was not measured, but it is considered to be near 100% due to the similarity to samples C and D. In samples E, F, and G, we confirmed that in each sample several NV centres have a *T*_2_ > 2.0 ms, while in the high-concentration sample H, *T*_2_ was <2.0 ms (see Supplementary Note 1). These results show the reproducibility of obtaining a long *T*_2_, and they support the existence of an optimum concentration region. It should be noted that the *T*_2_ of NV centres in samples grown under the same conditions but without phosphorus doping is shorter (*T*_2_ < 1 ms).

### Coherence times

**Fig. 2:**
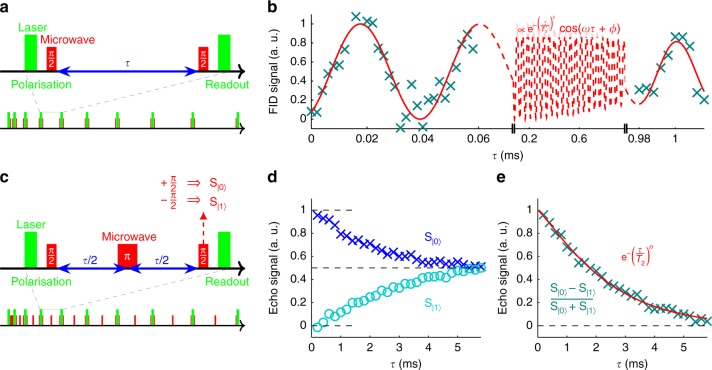
$T_2^ \ast$ and *T*_2_ in sample C. **a** Pulse sequence for a free-induction decay (FID) measurement, with a complete sequence illustrated at the bottom. **b** Result of FID measurement (data with blue crosses, sinusoidal exponential-decay fit with red line, $T_2^ \ast = 1.54_{ - 0.50}^{ + 1.91}$ ms). Please note the breaks on the horizontal axis; all data are fitted with a single function. **c** Pulse sequence for a Hahn-echo measurement. When applying a final +π/2-pulse, the spin is rotated towards the |0〉 state (*S*_|0〉_ measurement); for a final −π/2-pulse, the spin is rotated towards the |1〉 state (*S*_|1〉_ measurement). **d** Results for the *S*_|0〉_ measurement (blue crosses) and for the *S*_|1〉_ measurement (cyan circles). The top and bottom black dashed lines correspond to a maximum population of the |0〉 and |1〉 states, respectively. The middle black dashed line is when both states are populated equally. **e** Echo signal derived from subtracting **d**’s *S*_|1〉_ from **d**’s *S*_|0〉_ (data with blue crosses, exponential-decay fit with red line, $T_2 = 2.43_{ - 0.06}^{ + 0.06}$ ms). The dashed black line at 0 indicates when the states are populated equally

Owing to the pure NV^−^ state and long *T*_2_, sample C with [P] = 6 × 10^16^ atoms cm^−3^ was investigated more in depth. At first, $T_2^ \ast$ was studied^30^, measured via the exponential decay of a free-induction decay measurement (its well-known pulse sequence is illustrated in Fig. 2a). Since it is hard to measure $T_2^ \ast$ due to the strong effect of the environment, the measurement time is decreased by using both short *τ* and long *τ* in one measurement sequence (see Supplementary Note 2), resulting in $T_2^ \ast = 1.54_{ - 0.50}^{ + 1.91}$ ms (Fig. 2b). Compared with the previously reported long $T_2^ \ast$ of phosphor in an isotopically engineered ^28^Si crystal (270 μs^7^) and of NV centres in diamond (470 ± 100 μs^2^), this is the longest $T_2^ \ast$ for an electron spin ever observed in solid-state systems. The DC magnetic field sensitivity of the single NV centre can be derived to be ~6 nT Hz^−1/2^^13^.

To find NV centres with a long *T*_2_, Hahn-echo measurements (see Fig. 2c) were conducted for the ones with a long $T_2^ \ast$. Measurements for the |0〉 state (*S*_|0〉_: final π/2-pulse along the *x* axis, Fig. 2d) and the |1〉 state (*S*_|1〉_: final π/2-pulse along the −*x* axis, Fig. 2d) were subtracted and normalised as (*S*_|0〉_ − *S*_|1〉_)/(*S*_|0〉_ + *S*_|1〉_) to reject common-mode noise^20^, and the result was fitted to the exponential exp(−(*τ*/*T*_2_)^*n*^). Figure 2e shows that the longest *T*_2_ consistently measured $2.43_{ - 0.06}^{ + 0.06}$ ms, which is the longest *T*_2_ for an electron spin ever observed in solid-state systems at room temperature^3,19^. In these references, as opposed to our results, the measurements are performed without common-mode noise rejection, the results are rather noisy, and *n* was fixed at 2 for the fits. Hence, the uncertainty in *T*_2_ decreases (since *T*_2_ and *n* appear in the same exponent only), and the fitted *T*_2_ increases (since for long *T*_2_, generally *n* < 2; for our measurement, forcing *n* = 2 gives *T*_2_ = 2.93 ms). Moreover (see Fig. 1b), even the average 〈*T*_2_〉 = 1.8 ms of our measured NV centres rivals with the longest *T*_2_ measured in single-crystal diamond (*T*_2_ = 1.8 ms^3^), and almost 40% are longer than the longest *T*_2_ measured in poly-crystal diamond (*T*_2_ = 2.0 ms^19^), while both references only show their best measurement.

### Sensitivity

**Fig. 3:**
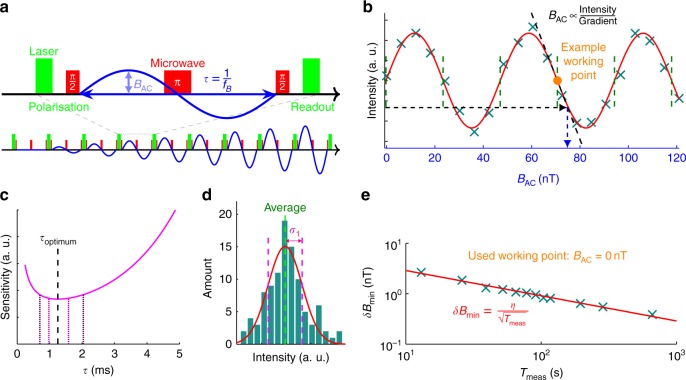
Alternating current (AC) magnetic field measurement. **a** Pulse sequence for a *B*_AC_ measurement. It is a Hahn-echo sequence (see Fig. 2c) with a synchronised sinusoidal AC magnetic field, which changes its sign during the π-pulse. Hence, the final phase depends on the magnetic field amplitude. A total sequence for a measurement like **b** is given at the bottom, where the amplitude of the magnetic field is increased at each Hahn-echo sub-sequence. The final microwave π/2-pulse is along the *y* axis, so that, at *B*_AC_ = 0 T, the gradient is at a maximum. **b** Single measurement to find the working points that have the maximum gradient (100,000 iterations, data with blue crosses, sinusoidal fit with red line). The working point indicated with an orange circle is an example, the dashed arrows show how a measured intensity translates to a magnetic field amplitude. **c** Theoretical sensitivity vs the time period of the magnetic field derived from the *T*_2_ data (see Supplementary Note 5), giving an optimum period *τ*_optimum_ (=1/*f*_*B*,optimum_) of 1.2 ms. The inner-magenta/outer-purple dotted vertical lines mark the range of periods for which the difference with the optimum sensitivity remains within 2%/10%, respectively. **d** Histogram of the repeatedly measured Hahn-echo intensity at working point *B*_AC_ = 0 T to determine the uncertainty *σ*_1_. For the vertical axis of **b** and the horizontal axis of **d**, the same units and scale are used (since the same analysis method is applied), so they cancel when computing *δB*_min_. **e** Logarithmic plot of *δB*_min_ vs *T*_meas_ (data with blue crosses, fit to *δB*_min_ = *η*/*T*_meas_ with red line, $\eta = 9.1_{ - 0.3}^{ + 0.3}$ nT Hz^−1/2^)

Since the AC magnetic field sensitivity is proportional to $1/\sqrt {T_2}$^31^, the NV centre with the longest *T*_2_ was examined. The concept for the measurement is given in Fig. 3a; the population of the spin state oscillates with the magnetic field amplitude (*B*_AC_). Therefore, to measure *B*_AC_, a working point of maximum gradient is chosen (the green dashed lines in Fig. 3b), and then the Hahn-echo intensity is measured. Via the gradient, this intensity relates directly to *B*_AC_. The amplitude of the sinusoidal magnetic field is derived from calibration with a DC magnetic field (see Supplementary Note 3).

The sensitivity is $\eta = \delta B_{{\mathrm{min}}}\sqrt {T_{{\mathrm{meas}}}}$ with *δB*_min_ the minimum detectable magnetic field amplitude, and *T*_meas_ the measurement time^31^. *δB*_min_ relates directly to the uncertainty of the measurement (see Supplementary Note 4) and is given by *δB*_min_ = *σ*_1_/grad with *σ*_1_ the uncertainty of a single Hahn-echo measurement and grad the gradient in the working point. Since *σ*_1_ depends on the noise in the system, which generally scales over measurement time with $1/\sqrt {T_{{\mathrm{meas}}}}$, the sensitivity is independent of the measurement time, hence its usefulness for comparing systems. Also, please be aware that the sensitivity of a measurement should be determined in the same way the measurement itself is conducted (see Supplementary Note 4), thus a technique such as used here is the only correct way to obtain a sensitivity.

Initially, the *T*_2_ data are used to estimate the optimum magnetic field frequency for the measurement. This optimum follows from the solution for *τ* in

1 $$\frac{{\tau + t_{{\mathrm{overhead}}}}}{\tau }n\left( {\frac{\tau }{{T_2}}} \right)^n - \frac{{t_{{\mathrm{overhead}}}}}{\tau } = \frac{1}{2},$$

with *τ* the time period of the magnetic field, *t*_overhead_ the overhead time of the pulse sequence, and *n* the power of the exponent from the fit to the *T*_2_ data. Its details and general results are explained in Supplementary Note 5. Figure 3c shows the solution for this NV centre, and although certain realistic circumstances that are not taken into account here (for example, temperature fluctuations) could influence the actual optimum given the flat area, it was opted to measure in the derived optimum point of *τ*_optimum_ ≈ 1.2 ms (hence *f*_AC_ ≈ 833 Hz).

To obtain the optimum sensitivity, first, the gradient in the working point was determined. Since this is a constant given the measurement parameters and environment, a relatively long time-averaging measurement can be used. An average result is shown in Fig. 3b, which gives grad = 1.56 × 10^7^ Intensity T^−1^. Next, the uncertainty of a measurement in the working point is extracted by measuring this point 100 times (Fig. 3d) for a number of measurement times *T*_meas_ ranging from 13 s to 11 min. In Fig. 3e, the resulting *δB*_min_ is fitted to $\eta /\sqrt {T_{{\mathrm{meas}}}}$, from which follows the sensitivity $\eta = 9.1_{ - 0.3}^{ + 0.3}$ nT Hz^−1/2^.

## Discussion

At first, the AC magnetic field sensitivity might seem worse than the previously reported best of a single NV centre at room temperature^3^. However, they used a different analysis method, which we investigated in order to compare (see Supplementary Note 6), which shows that we improved it by almost a factor of two. The reasons for this improvement are the longer *T*_2_, the larger Rabi contrast (see Fig. 1a)  and the higher photon count due to n-type diamond, and (to lesser account) the optimum sequence length (Supplementary Note 5). Please note that, although we demonstrated a synchronised measurement, other techniques are also limited by this optimal sensitivity, and hence any technique will have an improved sensitivity using our sample.

That the doping of phosphorus extends the spin-coherence times and gives better magnetic field sensitivities is against intuition, because phosphorus is paramagnetic at room temperature in diamond due to a large activation energy of 0.57 eV^28^ and thus causes magnetic noise. The time extensions in n-type diamond are considered to be due to charging of the vacancies, which suppresses the formation of paramagnetic vacancy complexes during growth. Potentially, this mechanism is similar to the recently reported interpretation of suppression of vacancy creation during ion-implantation through a sacrificial boron-doped p-type layer^22^. During the CVD growth, it is known that many vacancies are generated^21^, which causes generation of thermally stable impurity-vacancy and multi-vacancy complexes^16,32,33^. However, their generation can be suppressed by Coulomb repulsion of charged vacancies in n-type diamond, where an accepter level of the vacancy is located at 2.6 eV below the conduction band^34^, which is lower than the donor level of phosphorus (0.57 eV^28^).

**Fig. 4:**
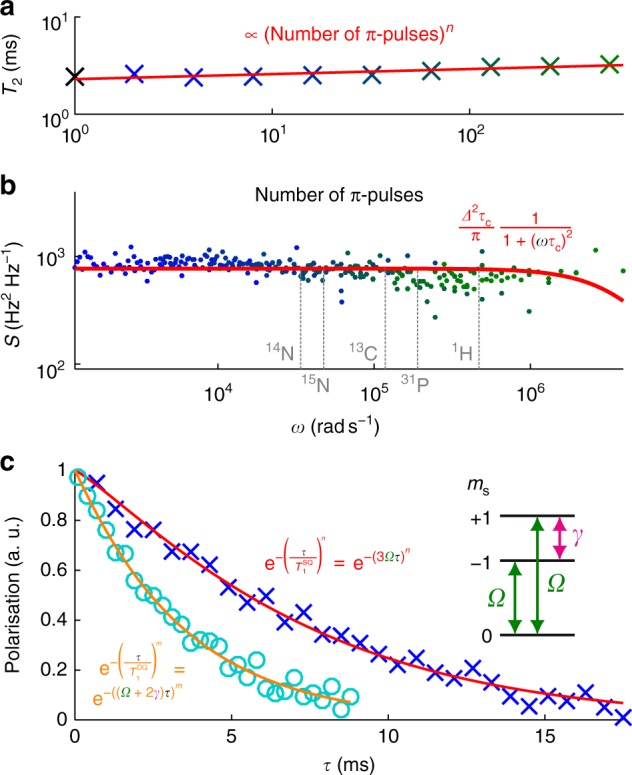
Noise analyses. **a** *T*_2_ vs number of π-pulses from measurements with Carr–Purcell–Meiboom–Gill sequences (datum with black cross for Hahn-echo, data with blue/green crosses for 2^1–9^ π-pulses, exponential fit with red line, exponent *n* = 0.05 ± 0.01). **b** Noise spectrum extracted from the same measurements as in **a** (starting from 2^1^ pulses, data with blue/green dots, Lorentzian fit with red line, correlation time *τ*_c_ fixed to represent the highest probed frequency). The Larmor frequencies of several nuclear spins are indicated (grey dashed lines); *B* = 1.8 mT. **c** Single quantum (data with blue crosses, exponential-decay fit with red line, measured $T_1^{{\mathrm{SQ}}}$ ≈ 7.4 ms) and double quantum (data with cyan circles, exponential-decay fit with orange line, measured $T_1^{{\mathrm{DQ}}}$ ≈ 3.4 ms) *T*_1_ measurements for estimating relaxation rates *Ω* (1/$T_1^{{\mathrm{SQ}}}$ = 3*Ω*) and *γ* (1/$T_1^{{\mathrm{DQ}}}$ = *Ω* + 2*γ*), which are illustrated in the inset

For a two-level system, *T*_2_ is known to be ultimately limited by the longitudinal spin-relaxation time *T*_1_. We applied Carr–Purcell–Meiboom–Gill (CPMG) dynamical-decoupling sequences^35–37^ with common-mode noise rejection, and *T*_2,dd_ = 3.3 ms was derived (for 512 π-pulses, see Fig. 4a). Although *T*_2,dd_ is longer than *T*_2_, it is still shorter than *T*_1_ (~6–7.5 ms^2,20^). To obtain information about the sources of decoherence, we carried out noise spectroscopy and *T*_1_ measurements.

The noise spectrum of deep NV centres can be analysed by a single Lorentzian^36^. For our data (see Fig. 4b), since the cut-off frequency eludes us, we fixed it as the highest probed frequency (thus *τ*_c_ ≤ 0.25 μs, the intracorrelation time of the bath spins) and fitted to find the minimum for *Δ* (*Δ* ≥ 0.1 MHz, the average coupling strength of the bath to the NV centre’s spin). The minimum density of the paramagnetic impurities/defects *n*_para_ was derived from *Δ* under the assumption that the noise source originates from dipolar interaction between these and the NV centre only (*n*_para_ ≈ *Δ*/*α* with *α* ≈ 3.3 × 10^−13^ s^−1^ cm^3^^31^), giving 3 × 10^17^ cm^−3^.

For a Lorentzian bath, in the limit of very short correlation times ($\tau _{\mathrm{c}} \ll T_2$), the dynamical-decoupling sequence is inefficient and there is no improvement with the number of pulses^36^. In addition, there is a limitation to the π-pulse duration, and their spacing restricts the maximum CPMG filter frequency^38^. We consider that these explain why the decoupling technique is not very effective for the extension of *T*_2_.

As for the contribution of nuclear spins, it is shown theoretically^39^ and experimentally^15^ that the concentration of nuclear spins inversely proportionally affects the *T*_2_ of the electron spin. From the extrapolation of their results for the concentration of nuclear spins in our sample, the contribution of the nuclear spin to the decoherence is considered to be very small. From the applied external magnetic field (1.8 mT), the Larmor frequencies of the nuclear spins of ^14^N, ^15^N, ^13^C, ^31^P, and ^1^H were calculated and they are indicated in Fig. 4b. They were not detected in the noise spectrum, which indicates as well that the contribution of the nuclear spins to the decoherence is small. For ^13^C, this is consistent with its expected density (0.002%).

Additionally, to infer an effect of electric-field noise, *T*_1_ is measured with single and double quantum measurements^38^. The relaxation rates *Ω* (between *m*_s_ = 0 and *m*_s_ = ±1, related to the magnetic noise) and *γ* (between *m*_s_ = −1 and *m*_s_ = +1, related to the electrical noise) were derived by fitting as shown in Fig. 4c. From fitting of the results of the NV centre that shows the longest *T*_2_, the rates are *Ω* ≈ 45 s^−1^ and *γ* ≈ 1.2 × 10^2^ s^−1^. In addition, we measured the rates of the NV centres that show shorter *T*_2_ in several phosphorus-doped samples (see Supplementary Note 7). In contrast to the previously reported rates for shallow NV centres^38^, they show 3*Ω* > *γ*, which indicates that magnetic noise is more prevalent in our samples, since for NV centres the actual single quantum 1/*T*_1_ = 3*Ω* + *γ*. Please note that this is the *T*_1_ limiting *T*_2_, which is actually just 3.9 ms for this NV centre, not to confuse with the *T*_1_ as given earlier (6 ms^20^, 7.5 ms^2^, and in our NV centre 7.4 ms), which means our *T*_2,dd_ is about 85% of the limiting *T*_1_.

In conclusion, in our phosphorus-doped n-type diamond sample, we were able to measure the longest $T_2^ \ast$ and *T*_2_, which leads to the best magnetic field sensitivities (among others such as temperature), which we confirmed for AC magnetic fields. The sensitivity improvements were not only due to the longer coherence times but also due to additional effects of our n-type diamond (increased Rabi contrast and photon count). From the above results, the main decoherence source is considered to be electron spins of impurities/defects. Analysing the noise spectrum estimates a minimum concentration of 3 × 10^17^ cm^−3^, which is larger than the phosphorus concentration. As shown in Fig. 1b, the longest measured *T*_2_ in diamond with [P] = 1 × 10^17^ atoms cm^−3^ is shorter than that in diamond with [P] = 6 × 10^16^ atoms cm^−3^, potentially because the phosphorus becomes the more dominant source of noise at higher concentrations. Therefore, elongation of *T*_2_ could be realised by optimising the phosphorus concentration and by continuing to decrease the paramagnetic impurities and defects. Thus our research opens a new avenue for further extension of coherence times of NV centres using new synthesis techniques of quantum-grade diamond. Moreover, the elongation of coherence times in n-type semiconductor diamond paves the way to the development and application of diamond-based quantum-information, sensing, and spintronics devices.

## Methods

### Phosphorus-doped diamond growth

The films of phosphorus-doped diamond were grown by MW plasma-enhanced CVD (PECVD) in a 5-kW magnetron generator equipped with a load-lock system. The base pressure of the main reactor was <2 × 10^−8^ Torr before CVD growth. Source gases were hydrogen (purity: N9), ^12^C-enriched methane (enrichment level: 99.998%, purified by a zirconium absorber), and phosphine (N6, dilution ratio PH_3_/H_2_ = 1000 ppm). The CH_4_/H_2_ gas flow rate was at a constant value of 0.4%, and the PH_3_/CH_4_ ratio was varied from 12.5 to 2000 ppm. The gas pressure, total gas flow, and MW power were 150 Torr, 1000 sccm, and 3600 W, respectively. During PECVD growth, the plasma and substrate temperatures were measured by optical pyrometry and confirmed to be stable. The incorporation of phosphorus was evaluated using secondary ion mass spectrometry. N-type conductivity, with a phosphorus donor level of 570 meV, and the electron mobilities were characterised by Hall-effect measurements. The electrodes were fabricated using the Van-der-Pauw contact configurations, and the details of the processes were the same as the ones reported previously. The mobilities depend on the phosphorus concentration and showed a scatter, but they were about 800 cm^2^ V^−1^ s^−1^ at 300 K, which are almost the same as the highest ones reported previously^28^.

## Supplementary information

Supplementary Information

Peer Review File

## Data Availability

The data that support the findings of this study are available from the corresponding author upon reasonable request.

## References

[1] Awschalom DD, Hanson R, Wrachtrup J, Zhou BB (2018). Quantum technologies with optically interfaced solid-state spins. Nat. Photonics.

[2] Maurer PC (2012). Room-temperature quantum bit memory exceeding one second. Science.

[3] Balasubramanian G (2009). Ultralong spin coherence time in isotopically engineered diamond. Nat. Mater..

[4] Christle DJ (2014). Isolated electron spins in silicon carbide with millisecond coherence times. Nat. Mater..

[5] Widmann M (2015). Coherent control of single spins in silicon carbide at room temperature. Nat. Mater..

[6] Kolesov R (2012). Optical detection of a single rare-earth ion in a crystal. Nat. Commun..

[7] Muhonen JT (2014). Storing quantum information for 30 seconds in a nanoelectronic device. Nat. Nanotechnol..

[8] Veldhorst M (2014). An addressable quantum dot qubit with fault-tolerant control-fidelity. Nat. Nanotechnol..

[9] Yoneda J (2018). A quantum-dot spin qubit with coherence limited by charge noise and fidelity higher than 99.9%. Nat. Nanotechnol..

[10] Hanson R, Awschalom DD (2008). Coherent manipulation of single spins in semiconductors. Nature.

[11] Zhu X (2011). Coherent coupling of a superconducting flux qubit to an electron spin ensemble in diamond. Nature.

[12] Kubo Y (2011). Hybrid quantum circuit with a superconducting qubit coupled to a spin ensemble. Phys. Rev. Lett..

[13] Dréau A (2011). Avoiding power broadening in optically detected magnetic resonance of single NV defects for enhanced DC magnetic field sensitivity. Phys. Rev. B.

[14] Isberg J (2002). High carrier mobility in single-crystal plasma-deposited diamond. Science.

[15] Mizuochi N (2009). Coherence of single spins coupled to a nuclear spin bath of varying density. Phys. Rev. B.

[16] Yamamoto T (2013). Extending spin coherence times of diamond qubits by high-temperature annealing. Phys. Rev. B.

[17] Stanwix PL (2010). Coherence of nitrogen-vacancy electronic spin ensembles in diamond. Phys. Rev. B.

[18] Jamonneau P (2016). Competition between electric field and magnetic field noise in the decoherence of a single spin in diamond. Phys. Rev. B.

[19] Jahnke KD (2012). Long coherence time of spin qubits in ^12^C enriched polycrystalline chemical vapor deposition diamond. Appl. Phys. Lett..

[20] Bar-Gill N, Pham LM, Jarmola A, Budker D, Walsworth RL (2013). Solid-state electronic spin coherence time approaching one second. Nat. Commun..

[21] Bar-Yam Y, Moustakas TD (1989). Defect-induced stabilization of diamond films. Nature.

[22] de Oliveira FF (2017). Tailoring spin defects in diamond by lattice charging. Nat. Commun..

[23] Doi Y (2016). Pure negatively charged state of the NV center in n-type diamond. Phys. Rev. B.

[24] Mizuochi N (2012). Electrically driven single-photon source at room temperature in diamond. Nat. Photonics.

[25] Lohrmann A (2011). Diamond based light-emitting diode for visible single-photon emission at room temperature. Appl. Phys. Lett..

[26] Bourgeois E (2015). Photoelectric detection of electron spin resonance of nitrogen-vacancy centres in diamond. Nat. Commun..

[27] Fukui N (2016). Ferromagnetic-resonance induced electromotive forces in Ni_81_Fe_19_ p-type diamond. Solid State Commun..

[28] Kato H, Ogura M, Makino T, Takeuchi D, Yamasaki S (2016). N-type control of single-crystal diamond films by ultra-lightly phosphorus doping. Appl. Phys. Lett..

[29] Waldherr G, Neumann P, Huelga SF, Jelezko F, Wrachtrup J (2011). Violation of a temporal Bell inequality for single spins in a diamond defect center. Phys. Rev. Lett..

[30] de Sousa R, Sarma SD (2003). Theory of nuclear-induced spectral diffusion: spin decoherence of phosphorus donors in Si and GaAs quantum dots. Phys. Rev. B.

[31] Taylor JM (2008). High-sensitivity diamond magnetometer with nanoscale resolution. Nat. Phys..

[32] Ammerlaan CAJ (1989). Paramagnetic centres in diamond, Landolt-Börnstein, New Series, Group III. Cryst. Res. Technol..

[33] Baker JM (2007). Deducing atomic models for point defects in diamond: the relevance of their mechanism of formation. Diam. Relat. Mater..

[34] Goss JP, Briddon PR, Jones R, Sque S (2004). Donor and acceptor states in diamond. Diam. Relat. Mater..

[35] Meiboom S, Gill D (1958). Modified spin-echo method for measuring nuclear relaxation times. Rev. Sci. Instrum..

[36] Bar-Gill N (2012). Suppression of spin-bath dynamics for improved coherence of multi-spin-qubit systems. Nat. Commun..

[37] Bylander J (2011). Noise spectroscopy through dynamical decoupling with a superconducting flux qubit. Nat. Phys..

[38] Myers BA, Ariyaratne A, Jayich ACB (2017). Double-quantum spin-relaxation limits to coherence of near-surface nitrogen-vacancy centers. Phys. Rev. Lett..

[39] Maze JR, Taylor JM, Lukin MD (2008). Electron spin decoherence of single nitrogen-vacancy defects in diamond. Phys. Rev. B.

