# Supplementary material for: Ultra-long coherence times amongst room-temperature solid-state spins
Source: Nat Commun. 2019 Aug 28;10:3766. doi: 10.1038/s41467-019-11776-8 (PMC6713727; doi:10.1038/s41467-019-11776-8)
Supplement: Supplementary file 1 — Supplementary Information [file 41467_2019_11776_MOESM1_ESM.pdf]

Supplementary information
Ultra-long coherence times amongst room-temperature solid-state spins

Herbschleb et al.

Contents

Supplementary Note 1: T_2 in phosphorus-doped samples E–H . . . 2
Supplementary Note 2: T_2^* measurement . . . 3
Supplementary Note 3: Magnetic field calibration . . . 5
Supplementary Note 4: Explanation of δB_{min} . . . 6
Supplementary Note 5: Optimum frequency for AC magnetic field measurement . . . 7
Supplementary Note 6: Comparison with current best AC magnetic field sensitivity . . . 10
Supplementary Note 7: Additional T_1 measurements . . . 11
Supplementary References . . . 12

Supplementary Note 1: T_2 in phosphorus-doped samples E–H

Supplementary Fig. 1 shows T_2s of NV centres in the samples E–H. Samples E–G have almost the same phosphorus concentrations as sample C in the main text, and sample H has a larger phosphorus concentration. It should be noted that the growth conditions of samples E–H are the same as those of samples A–D, except for the PH_3/CH_4 gas ratio to change the phosphorus concentration as described in the main text and in the Methods section. In samples E–G, we confirmed that several NV centres in each sample have a T_2 longer than 2.0 ms. In the high-concentration sample H, we found that T_2 was below 2 ms, which indicates again that an optimum exists.

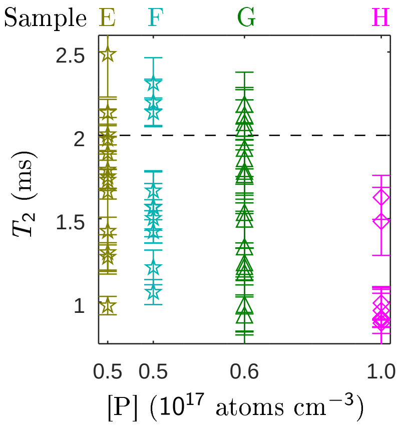

Supplementary Fig. 1. Measurement of T_2 for samples E–H. T_2 vs phosphorus concentration [P] for four samples E–H with similar concentrations as the samples used in the main text (Fig. 1b). Please note that the horizontal axis simply displays the phosphorus concentrations, and it is not linearly scaled. The error bars indicate standard errors.

Supplementary Note 2: T_2^* measurement

T_2^* follows from a free-induction decay measurement (FID)[1,2]. When detuning the frequency of the microwave field from the resonance frequency between the used energy levels of the NV centre, the phase of the electron spin rotates with this detuning frequency (in the rotational frame). The results are fitted to

$$A + B\mathrm{e}^{-\left(\frac{\tau}{T_2^*}\right)^n} \cos(\omega\tau + \phi), \tag{1}$$

with A and B constants, τ the delay, n the power of the exponent, ω the detuning frequency, and ϕ the phase (usually $-\pi$). Since the points that effect the power n are limited, the fitted n has a large uncertainty. To ensure fair comparison between NV centres, since n and T_2^* depend on each other, it was chosen to fix n at 2.

Roughly speaking, there are two different factors limiting the resulting T_2^*. The first are factors inside the sample, such as fluctuations in magnetic field created by nearby spins. The second are factors outside the sample related to the measurement setup, such as change of temperature which shifts the sample location and changes the magnetic field of the static magnet, and hence the magnetic field at the NV centre; and the $m_\mathrm{s} = 0$ energy level depends on temperature[3] as well. The former factors limit the actual T_2^*, and this is the T_2^* to measure. The latter factors limit the apparent T_2^*, but only due to external (in principle solvable without changing the sample) issues. For example, this is visible in a daily cycle of the detuning frequency (when keeping the microwave frequency the same), which might be related to the outside temperature.

To investigate the effect of this external additional change in detuning frequency, it was simulated numerically, an example is shown in Supplementary Fig. 2a. Supplementary Fig. 2b shows the effect of a frequency shift of 60 Hz min^{-1}, a fairly average rate during the day in our measurement setup, on the apparent T_2^* for different measurement times, for a number of actual T_2^*s. As expected, the longer the measurement, the worse the result. For measurements taking longer than 15 min, the actual T_2^* is getting rather obscured by the apparent T_2^*. The effect is stronger for a longer T_2^*.

In Supplementary Fig. 2c, the effect of different frequency shifts is simulated for a measurement time of 10 min. For shifts larger than about 80 Hz min^{-1}, the effect makes it difficult to differentiate between the actual T_2^*s, for longer T_2^* it becomes difficult with slower shifts already. Supplementary Fig. 2b and c are expected to look similar, since both the measurement time and the frequency shift affect the total change in detuning during the complete measurement.

There are several ways to deal with such slow changes. One way is to measure the average shift in the optically detected magnetic resonance (ODMR) spectrum[4] troughs' frequencies over time, and shift the frequency during the measurement accordingly. A second way is limiting the time of the measurement, since the shorter it is, the smaller the total shift. The latter method is shown in the paper, where the measurement has two parts. A relatively high detuning frequency is chosen, which is found by the first part of the pulse sequence, which uses short delays. This part takes relatively little time. The second part of the pulse sequence uses long delays, with enough points to capture the oscillation. Since the points in between are skipped, this limits the measurement time of the total sequence.

To find NV centres with a high potential to have a long actual T_2^*, first, short measurements (~5 min) with a gap of 0.5 ms are conducted. Since for long T_2^* the decay is rather small in these measurements, these only indicate whether their T_2^* is beyond 0.5 ms, an example is shown in Supplementary Fig. 2d. The most promising ones are measured with a longer gap, as shown in Supplementary Fig. 2e and f, and in the main text in Fig. 2b. However, the measurement time for a 1.0 ms gap is about 10 min, which means that the apparent T_2^* will become shorter (see Supplementary Fig. 2b). Therefore, although it is easy to find NV centres with results as in Supplementary Fig. 2d, extending the measurement time often reduces the apparent T_2^* to below 1.0 ms, even though the actual T_2^* is probably higher (see Supplementary Fig. 2b).

To conclude, the described effect in our measurement setup is given as example. This effect depends on many factors, such as the design of the experimental room and the season, and thus is different depending on the specific environment. For our setup in summer, the temperature oscillates with an amplitude of 1 K. When merely looking at the shift of the $m_\mathrm{s} = 0$ level by -74.2 kHz K^{-1} [3], this equals a change of ~150 kHz per ~12 h, thus a change of $\sim 2\times10^2$ Hz min^{-1}. This is larger than the 60 Hz min^{-1} used in the examples of Supplementary Fig. 2, since the actual shift is lower when closer to the more stable times of the day. Measurements were carried out around these stable times, to limit the effect as much as possible, which enables results as shown in Fig. 2b in the main text.

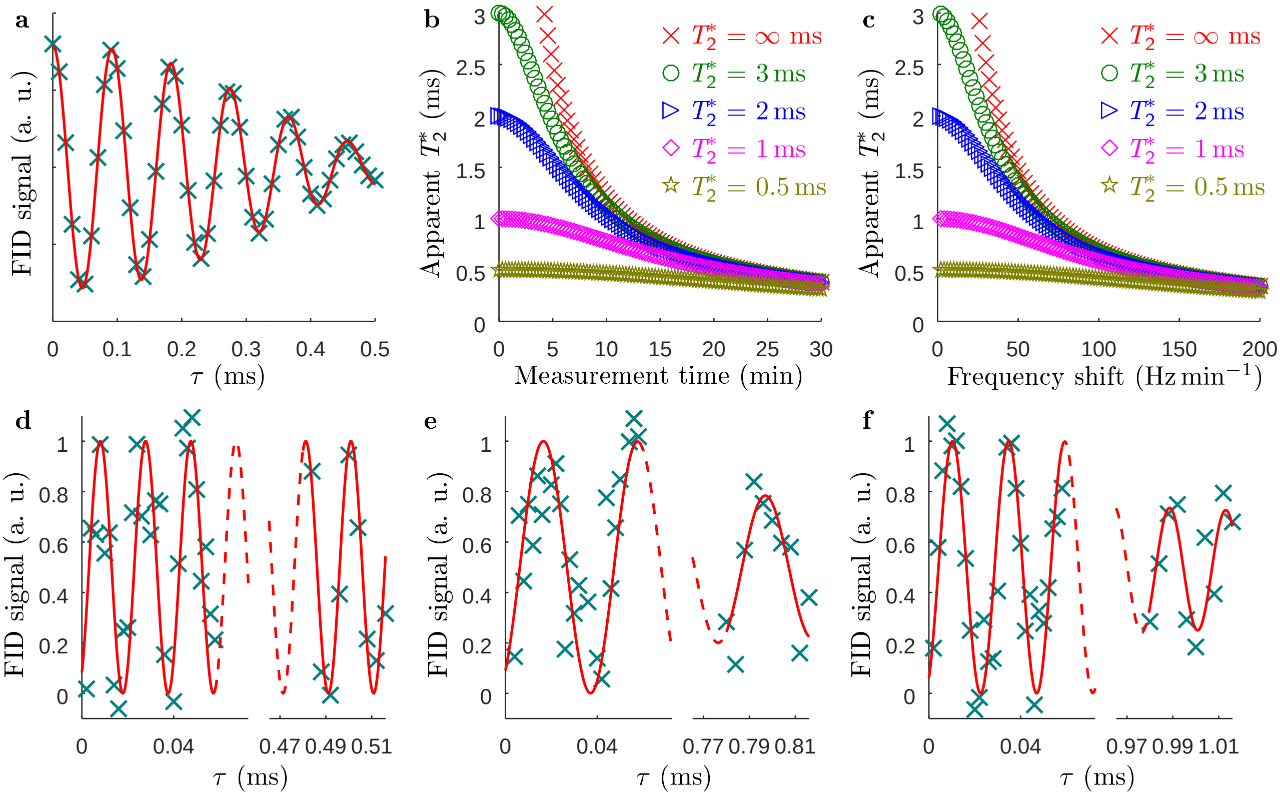

Supplementary Fig. 2. Simulation and measurement of T_2^*. **a** Simulation of the effect of a change in detuning frequency for a theoretical NV centre with $T_2^* = \infty$ s. The shift in frequency is 60 Hz min^{-1} (as measured during an average day in our setup), and the measurement time is 30 min (as for a similar non-gapped measurement with less points). The resulting apparent (thus fitted) $T_2^* = 0.39$ ms. **b** Simulated measured T_2^* vs measurement time for 5 different actual T_2^*s: ∞ ms (red crosses), 3 ms (green circles), 2 ms (blue triangles), 1 ms (magenta diamonds), and 0.5 ms (olive pentagrams). The simulated shift in frequency is 60 Hz min^{-1}. **c** Simulated measured T_2^* vs frequency shift over time for the same actual T_2^*s as in **b**. The simulated measurement time is 10 min. **d, e, f** Example results for gapped measurements (data with blue crosses, fits with red lines), please refer to **b** for the effect of longer measurements. **d** Gap of 0.5 ms, measurement time is 5 min, $T_2^* = 40,065^{+\infty}_{-40,064}$ ms. **e** Gap of 0.8 ms, measurement time is 8 min, $T_2^* = 1.1^{+2.6}_{-0.4}$ ms. **f** Gap of 1.0 ms, measurement time is 10 min, $T_2^* = 1.2^{+0.7}_{-0.4}$ ms.

Supplementary Note 3: Magnetic field calibration

When applying a magnetic field along the symmetry axis of the NV centre (here the z-axis), the $m_s = \pm 1$ energy levels are Zeeman split[5], with Zeeman energy

$$\Delta E_{\text{Zeeman}} = -\mu_z B_z = g\frac{e}{2m_e} S_z B_z = g\frac{e}{2m_e}\hbar B_z = g\mu_B B_z, \tag{2}$$

with μ_z the magnetic moment along the z-axis, B_z the magnetic field along the z-axis, g the g-factor of the electron spin, e the electron charge, m_e the mass of an electron, S_z the angular momentum along the z-axis, $\hbar$ reduced Planck's constant, and μ_B the Bohr magneton. Since $\Delta E = h\Delta f$, with h Planck's constant and Δf the shift in resonance frequency, the change in applied magnetic field amplitude follows from the locations of the troughs in the optically-detected magnetic resonance (ODMR) spectrum[4] as

$$\Delta B_z = \frac{h\Delta f}{g\mu_B}. \tag{3}$$

To calibrate the magnetic field induced by the coil near the sample, a series of DC voltages was applied to the coil, and the magnetic field amplitude was derived from the shift of the troughs in the ODMR spectrum. The result, plotted in Supplementary Fig. 3, is fitted to a first-order polynomial, from which the calibration factor follows ($|\delta B| = 12.10 \pm 0.05$ µT V^{-1}).

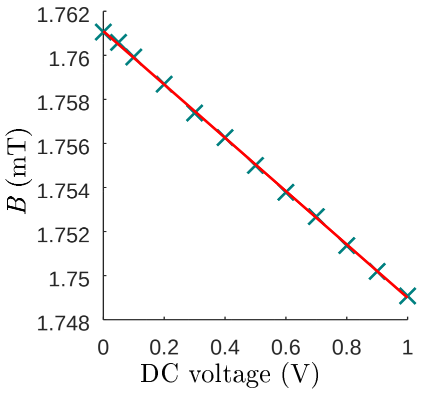

Supplementary Fig. 3. Magnetic field calibration. Magnetic field amplitude derived from the shift of the troughs in the ODMR spectrum vs the DC voltage applied to the coil (data with blue crosses, fit with red line).

Supplementary Note 4: Explanation of δB_{min}

To derive the minimum detectable magnetic field amplitude δB_{min}, first the magnetic field measurement itself needs to be defined. To measure the magnetic field amplitude, a Hahn-echo measurement is performed (see Fig. 3a in the main text). Then, the resulting intensity is converted into a magnetic field amplitude via their relationship (see Fig. 3b in the main text). This means, since this relation is sinusoidal, a certain working region around a working point needs to be chosen. The sensor would be most sensitive when the gradient (grad) in this point is largest. Thus, the initial system would be in one of these working points, then the magnetic field to measure should be applied, and after measuring the Hahn-echo intensity, the magnetic field can be computed.

For the AC magnetic field measurement, δB_{min} is the essential measure for its quality (the smaller the better). It is related to the uncertainty in the detected magnetic field amplitude. Given the uncertainty σ_1 of the measured variable (the intensity of a Hahn-echo measurement, as described above), the uncertainty in the magnetic field amplitude σ_B follows from the gradient grad, as illustrated in Supplementary Fig. 4a. This uncertainty is defined as the minimum detectable magnetic field amplitude, thus giving

$$\delta B_{\mathrm{min}} = \sigma_B = \frac{\sigma_1}{\mathrm{grad}}. \tag{4}$$

Please note that since this is merely a single standard deviation, for Gaussian variables, this only encompasses 38% of the results (so it has a 38% confidence interval), as shown in Supplementary Fig. 4b. Although this is not convincing as certainty (an 87% confidence interval, equivalent to $\delta B_{\mathrm{min}} = 3\sigma_B$, would be more appropriate), as long as the same definition is used (in others' experiments), the results can be compared.

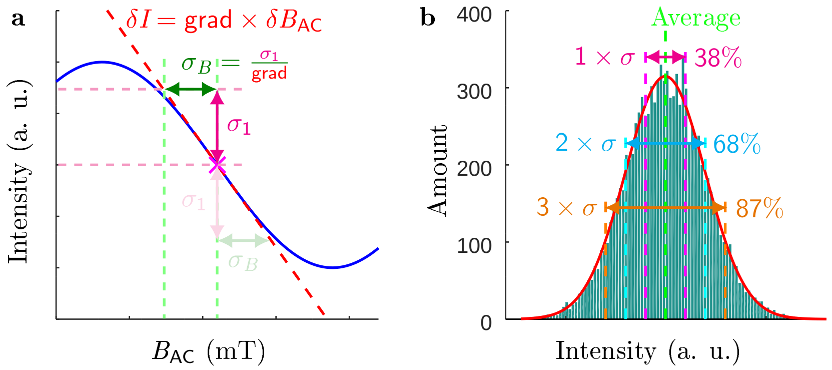

Supplementary Fig. 4. Origin of δB_{min}. **a** The uncertainty in magnetic field amplitude σ_B relates directly to the uncertainty in the Hahn-echo intensity σ_1 via the gradient grad at the working point. **b** Gaussian distribution with several standard deviations indicated: a single standard deviation σ includes 38% of the results (inner magenta dashed lines), two standard deviations 2σ include 68% of the results (middle cyan dashed lines), and three standard deviations 3σ include 87% of the results (outer orange dashed lines).

Supplementary Note 5: Optimum frequency for AC magnetic field measurement

Below, the optimum time period for the AC magnetic field measurement, and hence the frequency, is derived. The derivation starts with describing the sensitivity. Such descriptions exist, and are encouraged to read for comparison[6]. However, please note two important differences. The first, here, the focus is on calculating the optimum frequency. Secondly, the approach taken here is more experimentally oriented, while the formulae in for example[6] are more theoretically oriented.

The frequency of the magnetic field $f_B = 1/t$ that yields the lowest sensitivity is found as follows (definitions in Supplementary Fig. 5). The sensitivity η is defined by

$$\eta = \delta B_{\text{min}} \sqrt{T_{\text{meas}}} = \frac{\sigma_1}{\text{grad}_{\text{max}}} \sqrt{T_{\text{meas}}}, \tag{5}$$

where δB_{min} follows from Supplementary Note 4, and T_{meas} is the time it takes to measure the Hahn-echo intensity used to derive the magnetic field amplitude. In the shot-noise limit, the uncertainty of a single measurement of the Hahn-echo intensity σ_1 is (essentially a Poisson distribution divided by its mean)

$$\sigma_1 = \frac{1}{\sqrt{N_{\text{ph}} N}}, \tag{6}$$

with N_{ph} the average photon count per sequence, and N the number of iterations of the sequence. The latter is simply $N = \frac{T_{\text{meas}}}{t_{\text{sequence}}}$ with $t_{\text{sequence}} = t + t_{\text{overhead}}$ the time length of a single sequence, as shown in Supplementary Fig. 5.

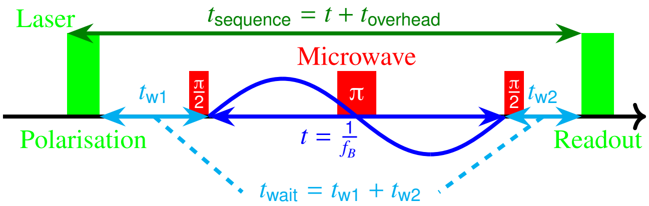

Supplementary Fig. 5. Definitions. Times used in the derivation of the optimum magnetic field frequency.

The maximum gradient grad_{max} follows from the sine-shaped fit ($A \sin(\omega B_{\text{AC}} + \phi) + S$) to the intensity vs magnetic field amplitude plot (see main text Fig. 3b), and is

$$\text{grad}_{\text{max}} = A\omega. \tag{7}$$

The amplitude A depends on the maximum contrast possible (for example measurable via Rabi oscillations) and the fraction of this maximum available, which depends on the t_{sequence} and T_2, since at $t_{\text{sequence}} \ll T_2$, the maximum contrast is reached, while at $t_{\text{sequence}} \gg T_2$, there is no contrast at all. Hence, the amplitude is given via the fit to the T_2 Hahn-echo data (Fig. 2e in the main text) as

$$A = D e^{-\left(\frac{t+t_{\text{wait}}}{T_2}\right)^n}, \tag{8}$$

with D a constant which is half the maximum contrast, and both T_2 and n follow from fitting the Hahn-echo data. Finally, the frequency ω (in magnetic field, so units are T^{-1}) follows from the accumulated phase $\Delta\phi$ due to the magnetic field. The latter stems from detuning due to the applied AC magnetic field, since the field shifts the resonance frequency (see Supplementary Note 3). The spin (in the rotational frame) rotates with the shift in detuning frequency Δf, hence the phase follows from integrating this frequency shift over the period between the $\pi/2$-pulses, taking into account the phase shift at the π-pulse halfway:

$$\Delta\phi = 2\pi \left[-\int_0^{\frac{\tau}{2}} \Delta f(t)\, dt + \int_{\frac{\tau}{2}}^{\tau} \Delta f(t)\, dt \right], \tag{9}$$

where τ is the delay time in the pulse sequence (between the two $\pi/2$-pulses). The detuning follows from Eq. (3). As can be seen from the integral, the effect of a DC magnetic field is cancelled due to the π-pulse. The effect of a sinusoidal AC magnetic field is

$$\begin{aligned} \Delta\phi &= 2\pi \left[-\int_0^{\frac{\tau}{2}} \frac{g\mu_{\text{B}} B_{\text{AC}}}{h} \sin(2\pi\nu t + \phi_{\text{AC}}) dt + \int_{\frac{\tau}{2}}^{\tau} \frac{g\mu_{\text{B}} B_{\text{AC}}}{h} \sin(2\pi\nu t + \phi_{\text{AC}}) dt \right] \\ &= 2\pi \frac{g\mu_{\text{B}} B_{\text{AC}}}{h} \frac{1}{2\pi\nu} \left[2\cos(\pi\nu\tau + \phi_{\text{AC}}) - \cos(\phi_{\text{AC}}) - \cos(2\pi\nu\tau + \phi_{\text{AC}}) \right], \end{aligned} \tag{10}$$

with g the g-factor of the electron spin, μ_B the Bohr magneton, B_{AC} the amplitude of the AC magnetic field, h Planck's constant, ν the frequency of the magnetic field (so units are s^{-1}), and ϕ_{AC} the phase of the magnetic field at the first $\pi/2$-pulse. With some trigonometry this can be reduced to

$$\Delta\phi = \frac{4g\mu_B B_{AC}}{h\nu}\sin^2\left(\frac{\pi\nu\tau}{2}\right)\cos(\pi\nu\tau + \phi_{AC}). \tag{11}$$

In the measurement, the magnetic field is synchronised ($\phi_{AC} = 0$), the delay time τ is given as t (see Supplementary Fig. 5), and the period of the AC magnetic field is chosen as the delay time (thus $\nu = 1/\tau = 1/t$). A full period in magnetic field happens every -2π of accumulated phase (it is effectively moving anticlockwise), therefore the period in magnetic field follows

$$\Delta\phi = -\frac{4g\mu_B B_{AC}}{h\frac{1}{t}} \overset{\Delta\phi=-2\pi}{\Longrightarrow} B_{AC,\,\text{period}} = \frac{2\pi h}{4g\mu_B t} \Longrightarrow \omega = \frac{2\pi}{B_{AC,\,\text{period}}} = \frac{4g\mu_B}{h}t. \tag{12}$$

Combining all above equations, the sensitivity is

$$\eta = \frac{\frac{1}{\sqrt{N_{ph}\frac{T_{meas}}{t+t_{overhead}}}}}{De^{-\left(\frac{t+t_{wait}}{T_2}\right)^n}\frac{4g\mu_B}{h}t}\sqrt{T_{meas}} = \frac{h}{4g\mu_B D\sqrt{N_{ph}}}\frac{\sqrt{t+t_{overhead}}}{te^{-\left(\frac{t+t_{wait}}{T_2}\right)^n}} = K\frac{\sqrt{t+t_{overhead}}}{te^{-\left(\frac{t+t_{wait}}{T_2}\right)^n}}. \tag{13}$$

For comparison, with $t_{overhead} = 0$, $\hbar = h/(2\pi)$, choosing $t = \alpha T_2$, and absorbing D, α, $\sqrt{N_{ph}}$ and the now-constant exponent into a constant C, the expression often used for the sensitivity given shot-noise[6,7] follows

$$\eta = \frac{\pi\hbar}{2g\mu_B C\sqrt{T_2}}, \tag{14}$$

where for example with 30% Rabi contrast, $N_{ph} \approx 0.03$[7] and $\alpha = 1$, $C \approx 0.3/2 \times \sqrt{0.03} \times e^{-1} \approx 0.01$.

To find the minimum sensitivity, the maximum of its inverse $1/\eta$ is determined by differentiation to t, and finding the zeros of the result. Performing the quotient and chain rules, left as exercise for the reader, the differentiation gives

$$\frac{\partial\frac{1}{\eta}}{\partial t} = \frac{1}{K}e^{-\left(\frac{t+t_{wait}}{T_2}\right)^n}\frac{\left[1-\frac{t}{t+t_{wait}}n\left(\frac{t+t_{wait}}{T_2}\right)^n\right]\sqrt{t+t_{overhead}} - \frac{1}{2}\frac{t}{\sqrt{t+t_{overhead}}}}{t+t_{overhead}}. \tag{15}$$

Equating this to zero, realising the exponent cannot be zero and the denominator is positive definite, the result for time t that gives the optimum time $t_{optimum}$ is (and $f_{B,\,\text{optimum}} = 1/t_{optimum}$)

$$\frac{t_{optimum}+t_{overhead}}{t_{optimum}+t_{wait}}n\left(\frac{t_{optimum}+t_{wait}}{T_2}\right)^n - \frac{t_{overhead}}{t_{optimum}} = \frac{1}{2}. \tag{16}$$

When using the same waiting time in the magnetic field pulse sequence as in the Hahn-echo sequence, effectively $t_{wait} = 0$ (since the fitting function for the Hahn-echo measurement used in Eq. (8) should include a $-t_{wait}$ as well, which is negligible for T_2 measurements). Finally, to get an idea of the result, without loss of generality, $t_{overhead} = \alpha t_{optimum}$ is chosen. Now, the solution to the above equation is

$$t_{optimum} = T_2\sqrt[n]{\frac{1+2\alpha}{2+2\alpha}\frac{1}{n}}. \tag{17}$$

For negligible overhead ($\alpha = 0$) and for extreme overhead ($\alpha = \infty$), the solutions are

$$t_{optimum} = \begin{cases} T_2\sqrt[n]{\frac{1}{2n}} & \text{for } t_{overhead} \ll t_{optimum} \\ T_2\sqrt[n]{\frac{1}{n}} & \text{for } t_{overhead} \gg t_{optimum}. \end{cases} \tag{18}$$

These solutions are plotted in Supplementary Fig. 6 as the fraction of T_2 vs the exponent of the Hahn-echo data n.

For quantum-projection noise limited measurements, the uncertainty σ_1 of a single measurement of the Hahn-echo intensity is

$$\sigma_1 = Y\frac{\sqrt{Np(1-p)}}{N} = Y\frac{1}{\sqrt{4N}}, \tag{19}$$

with N the number of iterations of the sequence, p the chance to measure the $|0\rangle$-state (1/2 at the maximum gradient), and Y a constant which depends on the contrast between the $|0\rangle$ and $|\pm1\rangle$ states. This has exactly the same shape as for the shot noise, hence the final result for the optimum frequency is the same. Analogue to Eq. (14), the sensitivity can be given as

$$\eta = \frac{\pi\hbar}{2g\mu_B\sqrt{T_2}}, \tag{20}$$

since Y from Eq. (19) and A from Eq. (8) cancel except for a factor of 2 ($Y = 2A$). Here, compared with shot noise (Eq. (14)), the sensitivity for projection noise is two orders of magnitude better.

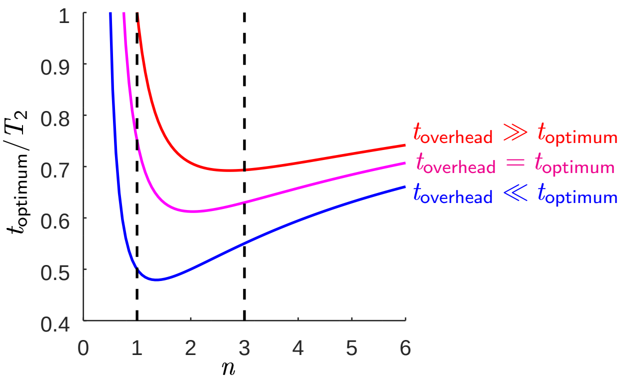

Supplementary Fig. 6. Optimum time period. The theoretical optimum time period $t_{optimum}$ (as fraction of T_2) for the AC magnetic field vs the exponent n of the T_2 data. The top red line is for $t_{overhead} \gg t_{optimum}$, the middle magenta line for $t_{overhead} = t_{optimum}$, and the bottom blue line for $t_{overhead} \ll t_{optimum}$. The black dashed vertical lines indicate the usual region for NV centres ($1 \leq n \leq 3$). For DC magnetic field measurements, the results are the same, with T_2^* instead of T_2.

For completeness, for the sensitivity of DC magnetic field measurements (using a FID measurement), the same procedure can be followed. Now, the phase is (equivalent to Eq. (9))

$$\Delta\phi = 2\pi \int_0^{\tau} \Delta f(t)\, dt = 2\pi \frac{g\mu_B}{h} B_{DC}\tau. \tag{21}$$

The frequency in magnetic field with measurement time $\tau = t$ (equivalent to Eq. (12)) is

$$\Delta\phi = 2\pi \frac{g\mu_B}{h} B_{DC} t \overset{\Delta\phi=2\pi}{\Longrightarrow} B_{DC,\, period} = \frac{h}{g\mu_B t} \Longrightarrow \omega = \frac{2\pi}{B_{DC,\, period}} = \frac{g\mu_B}{\hbar} t. \tag{22}$$

Combining this as in Eq. (13), with T_2^* instead of T_2, the sensitivity for DC fields is

$$\eta = \frac{\frac{1}{\sqrt{N_{ph}\frac{T_{meas}}{t+t_{overhead}}}}}{De^{-\left(\frac{t}{T_2^*}\right)^n}\frac{g\mu_B}{\hbar}t}\sqrt{T_{meas}} = \frac{\hbar}{g\mu_B D\sqrt{N_{ph}}}\frac{\sqrt{t+t_{overhead}}}{te^{-\left(\frac{t}{T_2^*}\right)^n}}. \tag{23}$$

Therefore, the optimum measurement time follows the same formula as the optimum time period for AC magnetic field measurements, and is shown in Supplementary Fig. 6.

Finally, with $t_{overhead} = 0$, choosing $t = \alpha T_2^*$, and absorbing D, α, $\sqrt{N_{ph}}$ and the now-constant exponent into a constant C, the expression often used for the sensitivity given shot-noise[6] follows

$$\eta = \frac{\hbar}{g\mu_B C\sqrt{T_2^*}}, \tag{24}$$

where for example with 30% Rabi contrast, $N_{ph} \approx 0.03$ and $\alpha = 1$, $C \approx 0.3/2 \times \sqrt{0.03} \times e^{-1} \approx 0.01$.

Supplementary Note 6: Comparison with current best AC magnetic field sensitivity

The current best sensitivity of AC magnetic field amplitudes with a single NV centre is 4.3 nT Hz$^{-1/2}$[8], their used NV centre has $T_2 \approx 1.8$ ms. Since the sensitivity $\eta \propto 1/\sqrt{T_2}$, our worse sensitivity is somewhat unexpected (9.1 nT Hz$^{-1/2}$), given our longer T_2 (2.4 ms). To compare, we looked in more detail at their paper, as described below.

In their paper, they determined the sensitivity in a different way: *"The sensitivity for a.c. magnetic field measurements was derived from the standard deviation of the fit function of experimental data points."* This means that the uncertainty of a single measurement σ_1, as explained in Supplementary Note 4, was estimated from the errors between the data and the fit (so with the residual norm) as

$$\sigma_1 \approx \frac{\text{residual norm}}{\sqrt{\#\text{points} - 1}} = \frac{\sqrt{\sum_{i=0}^{\#\text{points}-1} (f_i - F_i)^2}}{\sqrt{\#\text{points} - 1}} = \sqrt{\frac{1}{\#\text{points} - 1} \sum_{i=0}^{\#\text{points}-1} (f_i - F_i)^2}, \tag{25}$$

with f_i the measured data point, and F_i the value of the fit at that data point. Since this assumes that the noise is similar at the maximum gradient and at the crests and troughs (it is potentially larger at the maximum gradient due to the larger effect of potential magnetic noise, and the larger effect, if significant, of quantisation noise), and since this relies on a single measurement, the resulting rough estimate for the standard deviation is likely smaller than the one determined in an actual measurement scenario. However, as long as an average measurement is used (so not the best one, since noise is random and can be below average), the resulting estimate for the sensitivity is still acceptable.

To compare, we applied the same analysis method to our data from Fig. 3b in the main text, which gives $\eta = 8.6$ nT Hz$^{-1/2}$. As expected, this rough estimate gives a somewhat better sensitivity than the correctly measured result from the main text.

This is still significantly worse than the current best sensitivity. To clarify the difference, the data were extracted from their paper (Fig. 4b in their paper[8]) and their technique was applied. Since both the error of the fit and the maximum gradient are affected equally, the scale and units of the intensity do not matter, because they cancel when computing δB_{min} (see Eq. (4)). The minimum sequence length t_{sequence} (which gives the best sensitivity) follows from the magnetic field frequency ($t_{\text{sequence}} = 1.2$ ms, see Eq. (12)). The measurement time T_{meas} was not mentioned in the paper, so it is estimated from the shot noise (Eq. (6)), which they say is their limit. The photon count per sequence is estimated to be similar as the paper with the previous best sensitivity one year earlier[7] ($N_{\text{ph}} = 0.03$). With scaling of their result to the expected contrast given T_2 and the maximum Rabi contrast of 30% (resulting in a contrast of $0.3\mathrm{e}^{-\left(\frac{t_{\text{sequence}}}{T_2}\right)^2}$), $N \approx 1.0 \times 10^5$. Combining all data we get

$$\eta \approx \frac{\text{residual norm}}{\sqrt{\#\text{points} - 1}} \frac{1}{\text{grad}_{\text{max}}} \sqrt{N \times t_{\text{sequence}}} \approx 15 \text{ nT Hz}^{-1/2}. \tag{26}$$

This result, as opposed to the reported 4.3 nT Hz$^{-1/2}$, is what we would expect, given our longer T_2, our higher Rabi contrast and the potentially higher photon count in our n-type sample[9], and their non-optimum sequence length (see Supplementary Note 5). Also, compared with the previous best sensitivity[7] ($T_2 = 676$ μs, $\eta \approx 30$ nT Hz$^{-1/2}$), their almost threefold improvement of T_2 to 1.8 ms would imply a $\sim \sqrt{3}$ improvement of the sensitivity, thus giving $\eta \approx 18$ nT Hz$^{-1/2}$, close to what we computed above.

Since $N_{\text{ph}} = 0.03$ is an assumption (although fair), we also look at the best-case scenario, in which the assumption is that their setup was of similar standard as a more recent result from one of their groups[10], which gives the saturation intensity (252 kcs^{-1}). Therefore, the maximum photon count per sequence can be derived ($N_{\text{ph}} \leq 0.076$). (Please note that this is for the case that the spin state $m_s = 0$, but for the magnetic measurements it is in the superposition $1/\sqrt{2}\,(|0\rangle + |1\rangle)$ between the spin states, which with a Rabi contrast of 30% gives $N_{\text{ph}} \leq 0.064$.) Using the maximum, the sensitivity is $\eta \approx 10$ nT Hz$^{-1/2}$, which is still worse than our result from the main text. Although we do not know the reason for the difference between their reported value and our computed value, please note that this does not change their main results: their main message of using isotopically purified ^{12}C to improve T_2 is unchanged, and moreover, 15 nT Hz$^{-1/2}$ is still a (realistic) improvement over the previous best sensitivity[7].

To conclude, we compute our own experimentally achievable best sensitivity. In our case, we can simply infer the number of photons from our measurement directly, and it is $N_{\text{ph}} \approx 0.07$ (thus this is for the superposition). With our Rabi contrast of ~36%, and a minimum power of the exponent of 1 (due to the almost constant noise spectrum), the constant $C \approx 0.36/2 \times \sqrt{0.07} \times \mathrm{e}^{-\left(\frac{1.2e-3}{2.43e-3}\right)^1} \approx 0.029$, and hence the experimentally achievable best sensitivity is $\eta \approx 8.9$ nT Hz$^{-1/2}$, which is better than our result from the main text (as it should be).

Supplementary Note 7: Additional T_1 measurements

Additional T_1 data are given in Supplementary Table 1. Please note that, just like the data in the main text, these data are measured with common-mode noise rejection. However, less iterations are used, hence the results have larger uncertainties, nonetheless a similar trend for the rates seems rather likely ($3\Omega > \gamma$).

[phosphorus] (atoms cm^{-3})	1×10^{16}	1×10^{16}	1×10^{16}	6×10^{16}	1×10^{17}	1×10^{17}
NV^- population (%)	79	91	93	100	100	100
T_2 (ms)	1.3	1.7	2.0	1.9	1.2	1.8
T_1^{SQ} (ms)	5.6	7.4	8.2	7.2	5.9	7.9
T_1^{DQ} (ms)	4.3	5.7	3.7	3.3	3.3	3.9
Ω (s^{-1})	59	45	41	46	56	42
γ (s^{-1})	88	66	1.1×10^2	1.3×10^2	1.2×10^2	1.1×10^2

Supplementary Table 1. T_1 measurements. T_1 measured for several NV centres in different samples.

Supplementary References

1. de Sousa, R. & Sarma, S. D. Theory of nuclear-induced spectral diffusion: spin decoherence of phosphorus donors in Si and GaAs quantum dots. *Phys. Rev. B* **68**, 115322 (2003).

2. Maze, J. R. *et al.* Free induction decay of single spins in diamond. *New J. Phys.* **14**, 103041 (2012).

3. Acosta, V. M. *et al.* Temperature dependence of the nitrogen-vacancy magnetic resonance in diamond. *Phys. Rev. Lett.* **104**, 070801 (2010).

4. Gruber, A. *et al.* Scanning confocal optical microscopy and magnetic resonance on single defect centers. *Science* **276**, 2012–2014 (1997).

5. Zeeman, P. Over doubletten en tripletten in het spektrum, teweeggebracht door uitwendige magnetische krachten. *Versl. Kon. Akad. Wetensch. Amsterdam* **6**, 13–18, 99–102, 260–262 (1897).

6. Taylor, J. M. *et al.* High-sensitivity diamond magnetometer with nanoscale resolution. *Nat. Phys.* **4**, 810–816 (2008).

7. Maze, J. R. *et al.* Nanoscale magnetic sensing with an individual electronic spin in diamond. *Nature* **455**, 644–647 (2008).

8. Balasubramanian, G. *et al.* Ultralong spin coherence time in isotopically engineered diamond. *Nat. Mater.* **8**, 383–387 (2009).

9. Doi, Y. *et al.* Pure negatively charged state of the NV center in n-type diamond. *Phys. Rev. B* **93**, 081203 (2016).

10. Rogers, L. J. *et al.* Multiple intrinsically identical single-photon emitters in the solid state. *Nat. Commun.* **5**, 4739 (2014).
