# Supplementary material for: Ultra-long coherence times amongst room-temperature solid-state spins
Source: Nat Commun. 2019 Aug 28;10:3766. doi: 10.1038/s41467-019-11776-8 (PMC6713727; doi:10.1038/s41467-019-11776-8)
Supplement: Supplementary file 2 — Peer Review File [file 41467_2019_11776_MOESM2_ESM.pdf]

Editorial Note: This manuscript has been previously reviewed at another journal that is not operating a transparent peer review scheme. This document only contains reviewer comments and rebuttal letters for versions considered at Nature Communications.

REVIEWERS' COMMENTS:

Reviewer #2 (Remarks to the Author):

The authors have responded well to the criticism raised by the referees and significantly improved the paper. Most of the remaining open points are left open on the basis that they would require too much work and would therefore go beyond the present paper. I therefore feel that this version of the paper should be published.
Dieter Suter

Reviewer #3 (Remarks to the Author):

The authors have adequately addressed my comments and questions. They have also made considerable revisions to address the comments from other reviewers. I recommend the publication of the manuscript in Nature Communications.
